# Manipulation of isolated brain nerve terminals by an external magnetic field using D-mannose-coated γ-Fe_2_O_3_ nano-sized particles and assessment of their effects on glutamate transport

**DOI:** 10.3762/bjnano.5.90

**Published:** 2014-06-04

**Authors:** Tatiana Borisova, Natalia Krisanova, Arsenii Borуsov, Roman Sivko, Ludmila Ostapchenko, Michal Babic, Daniel Horak

**Affiliations:** 1 The Department of Neurochemistry, Palladin Institute of Biochemistry, NAS of Ukraine, 9 Leontovicha Street, Kiev, 01601, Ukraine; 2 The Biological Faculty, Taras Shevchenko National University of Kyiv, 64 Volodymyrska Str, Kiev, Ukraine; 3 The Department of Polymer Particles, Institute of Macromolecular Chemistry AS CR, Heyrovsky Sq. 2, 162 06 Prague 6, Czech Republic

**Keywords:** extracellular level, γ-Fe_2_O_3_, glutamate uptake and release, manipulation by an external magnetic field, D-mannose, membrane potential, nanoparticles, rat brain nerve terminals, synaptic vesicle acidification

## Abstract

The manipulation of brain nerve terminals by an external magnetic field promises breakthroughs in nano-neurotechnology. D-Mannose-coated superparamagnetic nanoparticles were synthesized by coprecipitation of Fe(II) and Fe(III) salts followed by oxidation with sodium hypochlorite and addition of D-mannose. Effects of D-mannose-coated superparamagnetic maghemite (γ-Fe_2_O_3_) nanoparticles on key characteristics of the glutamatergic neurotransmission were analysed. Using radiolabeled L-[^14^C]glutamate, it was shown that D-mannose-coated γ-Fe_2_O_3_ nanoparticles did not affect high-affinity Na^+^-dependent uptake, tonic release and the extracellular level of L-[^14^C]glutamate in isolated rat brain nerve terminals (synaptosomes). Also, the membrane potential of synaptosomes and acidification of synaptic vesicles was not changed as a result of the application of D-mannose-coated γ-Fe_2_O_3_ nanoparticles. This was demonstrated with the potential-sensitive fluorescent dye rhodamine 6G and the pH-sensitive dye acridine orange. The study also focused on the analysis of the potential use of these nanoparticles for manipulation of nerve terminals by an external magnetic field. It was shown that more than 84.3 ± 5.0% of L-[^14^C]glutamate-loaded synaptosomes (1 mg of protein/mL) incubated for 5 min with D-mannose-coated γ-Fe_2_O_3_ nanoparticles (250 µg/mL) moved to an area, in which the magnet (250 mT, gradient 5.5 Т/m) was applied compared to 33.5 ± 3.0% of the control and 48.6 ± 3.0% of samples that were treated with uncoated nanoparticles. Therefore, isolated brain nerve terminals can be easily manipulated by an external magnetic field using D-mannose-coated γ-Fe_2_O_3_ nanoparticles, while the key characteristics of glutamatergic neurotransmission are not affected. In other words, functionally active synaptosomes labeled with D-mannose-coated γ-Fe_2_O_3_ nanoparticles were obtained.

## Introduction

Nanoparticles have great biotechnological potential opening a wide range of new applications. Properties of nanomaterials often differ from those in bulk forms providing unexpected physical and chemical properties. Therefore, a detailed understanding of principles of nanoparticle interaction with the cells is critical. Regarding the central nervous system, the investigation of the interaction of nanoparticles with neurons showed both negative and positive effects [1]. One of the concerns is that nanoparticles can potentially harm the function of or have toxic effects on human nerve cells owing to their ability to pass through biological membranes [2].

Superparamagnetic iron oxide nanoparticles are considered as promising candidates to increase the efficiency of targeted drug delivery not because of the possibility to attach antibodies to their surfaces, but also because of the possibly to use external magnetic guidance [3]. Superparamagnetism is an important characteristic because when the external magnetic field is taken away, the inner magnetization of nanoparticles disappears, and therefore their agglomeration, which carries the risk of embolization of the capillary vessels, can be avoided [3]. A key issue for enhancing of permeability of iron oxide nanoparticles through the cell membrane is the modification of their surface. In this context, biocompatible polymers can be attached to the surface of the nanoparticles to avoid their agglomeration and enhance their non-specific intracellular uptake [4]. Magnetic resonance imaging could be used for tracking labeled cells in vivo by using iron oxide nanoparticles coated by dextran [5–6]. Recently, immortalized cells of the MHP36 hippocampal cell line labeled with gadolinium rhodamine dextran in vitro were tracked in ischemia-damaged rat hippocampus in perfused brains ex vivo [7]. Contrast agents based on dextran-coated iron oxides are commercially available as blood pool agents for human use (Endorem, Guerbet, France; Resovist, Bayer Schering Pharma AG, Germany). Until the discontinuation of their manufacture, they had been used for study of migration of Endorem-labeled cells to a cortical photochemical lesion or compression of a spinal cord lesion in rats [8–9].

Some cells possess receptors for D-mannose on their membranes, that is, MMR on dendritic cells subsets, macrophages, lymphatic and hepatic endothelium, Endo 180 on subsets of endothelial cells, DC-SIGNR on hepatic and lymphatic endothelial cells as well as serum contains mannose binding lectines (MBL) [10]. In addition, D-mannose-bound poly(2-hydroxyethyl methacrylate) hydrogels support the adhesion of keratinocytes (epidermal cells) and their subsequent cultivation in the absence of feeder cells [11]. Therefore, D-mannose was selected to coat the iron oxide nanoparticles. Recently, it was demonstrated that D-mannose-modified iron oxide nanoparticles could cross the cell membranes and be internalized by rat bone marrow stromal cells [12].

In the mammalian central nervous system, amino acid glutamate plays a primary role as a key excitatory neurotransmitter. Glutamate participates in many aspects of normal brain functioning. Impaired glutamate homeostasis causes neuronal dysfunction and contributes to the pathogenesis of major neurological disorders. For normal brain functioning, a low extracellular level of glutamate should be maintained between episodes of exocytotic release, thereby preventing continual activation of glutamate receptors and protecting neurons from excitotoxic injury [13]. A certain glutamate concentration in the synaptic cleft is kept by high-affinity Na^+^-dependent glutamate transporters located in the plasma membrane of neurons and glial cells. For the uptake of neurotransmitters, glutamate transporters use Na^+^/K^+^ electrochemical gradients across the membrane as a driving force. As glutamate transporters are integral membrane proteins, it is clear that their functioning is closely associated with the plasma membrane, and so consequently can be influenced by changes in its properties. Synaptic vesicles, which are acidic compartments of nerve terminals, store neurotransmitters and release their contents by exocytosis upon stimulation. Acidification of synaptic vesicles accompanied by loading of the neurotransmitters appears to be their common property [14]. The active transport of not only glutamate, but also acetylcholine, monoamines, and γ-aminobutyric acid/glycine to synaptic vesicles is accomplished by vesicular transporters of the neurotransmitters, whose function depends on the proton electrochemical gradient Δμ_H+_ generated by V-ATPase that pumps protons into the vesicle interior.

The aim of the study was to assess the ability of new D-mannose-coated γ-Fe_2_O_3_ nanoparticles to move isolated brain nerve terminals in response to application of an external magnetic field and to analyze their effect on key characteristics of glutamatergic neurotransmission: (1) the uptake of glutamate by rat brain nerve terminals via specific high-affinity Na^+^-dependent plasma membrane transporters by using radiolabeled L-[^14^C]glutamate; (2) the membrane potential (*E*_m_) of the plasma membrane of nerve terminals by using potential sensitive fluorescent dye rhodamine 6G; and (3) the acidification of synaptic vesicles in nerve terminals by using pH-sensitive fluorescent dye acridine orange.

## Results

### D-Mannose-coated superparamagnetic γ-Fe_2_O_3_ nanoparticles: Synthesis and characterization

In this paper, superparamagnetic iron oxide nanoparticles were synthesized by the well-known precipitation of iron salts, namely FeCl_2_ and FeCl_3_, by rapid increase of pH by ammonia. Similarly as described in [12], this was followed by the oxidation of the resulting magnetite (Fe_3_O_4_) with sodium hypochlorite producing maghemite (γ-Fe_2_O_3_), which is chemically more stable than Fe_3_O_4_. This is in contrast to magnetite, which undergoes spontaneous oxidation by air. Proof of the maghemite by Mössbauer spectroscopy and powder X-ray diffraction, as well as its magnetic properties were described in our previous reports [15–16]. The neat γ-Fe_2_O_3_ nanoparticles were used in control experiments with cells or were used as a base for the subsequent modification with D-mannose, thus hindering the agglomeration of the particles when stored for long time.

**Figure 1:**
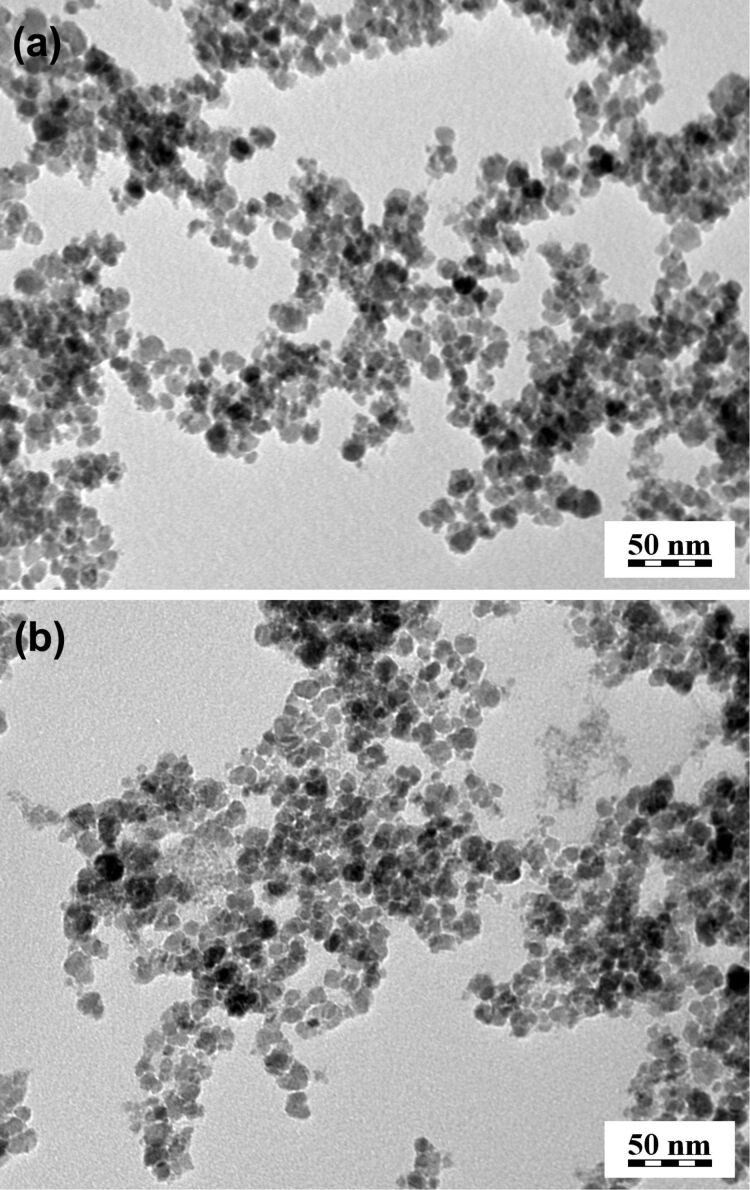
Transmission electron micrographs of (a) neat and (b) D-mannose-coated γ-Fe_2_O_3_ nanoparticles.

The coating of iron oxide colloidal nanoparticles was achieved by a post-synthesis modification with D-mannose [12]. D-mannose-coated nanoparticles were prepared at a D-mannose/γ-Fe_2_O_3_ ratio of 5.8:1 (w/w). According to TEM, the size of both neat and coated nanoparticles was about 7 nm and the polydispersity index PDI of about 1.4 (PDI = *D*_w_/*D*_n_, where *D*_w_ is the weight-average particle diameter, *D*_w_ = Σ *N*_i_* D*_i_^4^/Σ *N*_i_* D*_i_^3^ and *D*_n_ = Σ *N*_i_* D*_i_/Σ *N*_i_, where *N*_i_ is the number of particles with the diameter *D*_i_) documents a moderately broad particle size distribution (Table 1). The obtained iron oxide nanoparticle colloids were also investigated by dynamic light scattering (DLS). The hydrodynamic diameter (*D*_h_) of the nanoparticles calculated from DLS was about 10 times larger than the number-average diameter (*D*_n_) from TEM (Table 1) due to different evaluation statistics used. While *D*_n_ was calculated as a number average of manually measured particle diameters in a TEM micrograph (Figure 1), the hydrodynamic diameter was calculated by the method of cumulants [17]. In fact, the hydrodynamic diameter is closely related to the z-average size (*D*_z_ ≈ ∑*D*_i_^6^/∑*D*_i_^5^), the calculation of which favors large particles in comparison with the number-average diameter, where the diameter is in the first power. The polydispersity, PI, of both initial and coated particles determined from DLS was about 0.17 suggesting that the particle size distribution was not too broad, which is in agreement with the TEM analysis. Particle diameters in Table 1 thus do not show any significant differences between neat and D-mannose-coated γ-Fe_2_O_3_. It can be therefore concluded that the observed effects of D-mannose coating in biological experiments can be ascribed to chemical properties of the D-mannose, while the effects of physical parameters can be neglected.

**Table 1:** Properties of superparamagnetic nanoparticles.^a^

sample	*D*_n_ (nm)	PDI	*D*_h_ (nm)	PI

neat γ-Fe_2_O_3_	6.6	1.38	88.6 ± 0.7	0.162 ± 0.006
D-mannose-coated γ-Fe_2_O_3_	6.8	1.42	88.3 ± 0.04	0.17 ± 0.01

^a^*D*_n_: number-average diameter; PDI: polydispersity index, *D*_h_: hydrodynamic diameter, PI: polydipersity from DLS.

**Figure 2:**
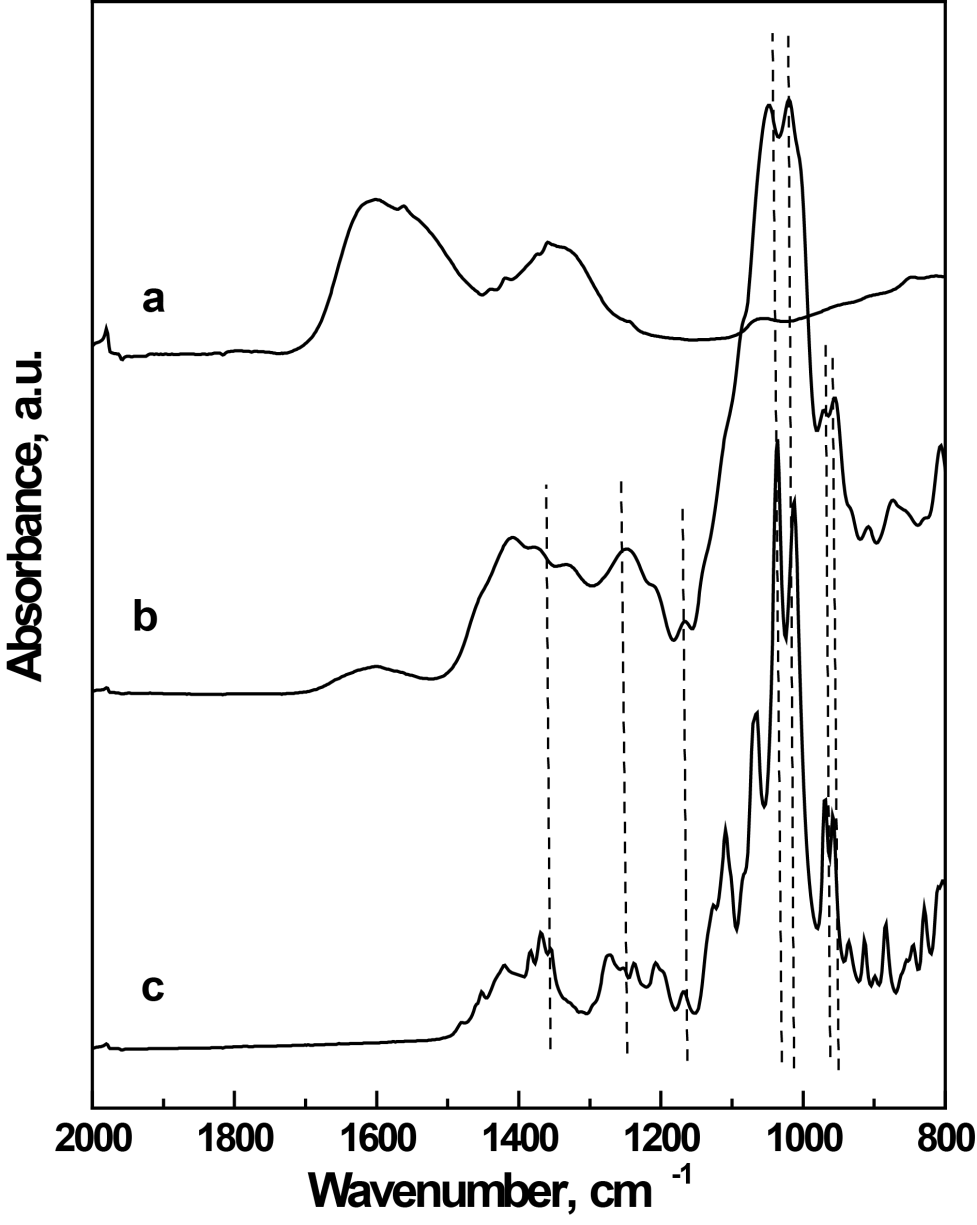
ATR-FTIR spectra of γ-Fe_2_O_3_ particles before (a) and after (b) coating with D- mannose. Spectrum (c) belongs to pure D-mannose.

Similarly as described in [12], the composition of the surface of nanoparticles was investigated by using ATR FTIR spectroscopy. The spectra of iron oxide before coating and after modification with D-mannose and the spectrum of D-mannose are displayed in Figure 2. Rather large iron oxide peaks were notable in the spectrum of the neat magnetic particles (Figure 2a). The characteristic bands of the C–H, C–O–H and C–O–C vibrations of D-mannose (Figure 2c) were apparent in the spectrum of the surface-modified magnetic nanoparticles (Figure 2b). This suggests that the surface of the γ-Fe_2_O_3_ particles was coated with D-mannose (Figure 2). D-mannose may be bonded to the surface of the particles via the hydroxy group located on the C2 carbon in the axial position. This is a configuration specific to D-mannose, in contrast to glucose and other common sugars, which have this hydroxy group only in the equatorial position.

### Glutamate uptake by nerve terminals in the presence of D-mannose-coated γ-Fe_2_O_3_ nanoparticles

**Figure 3:**
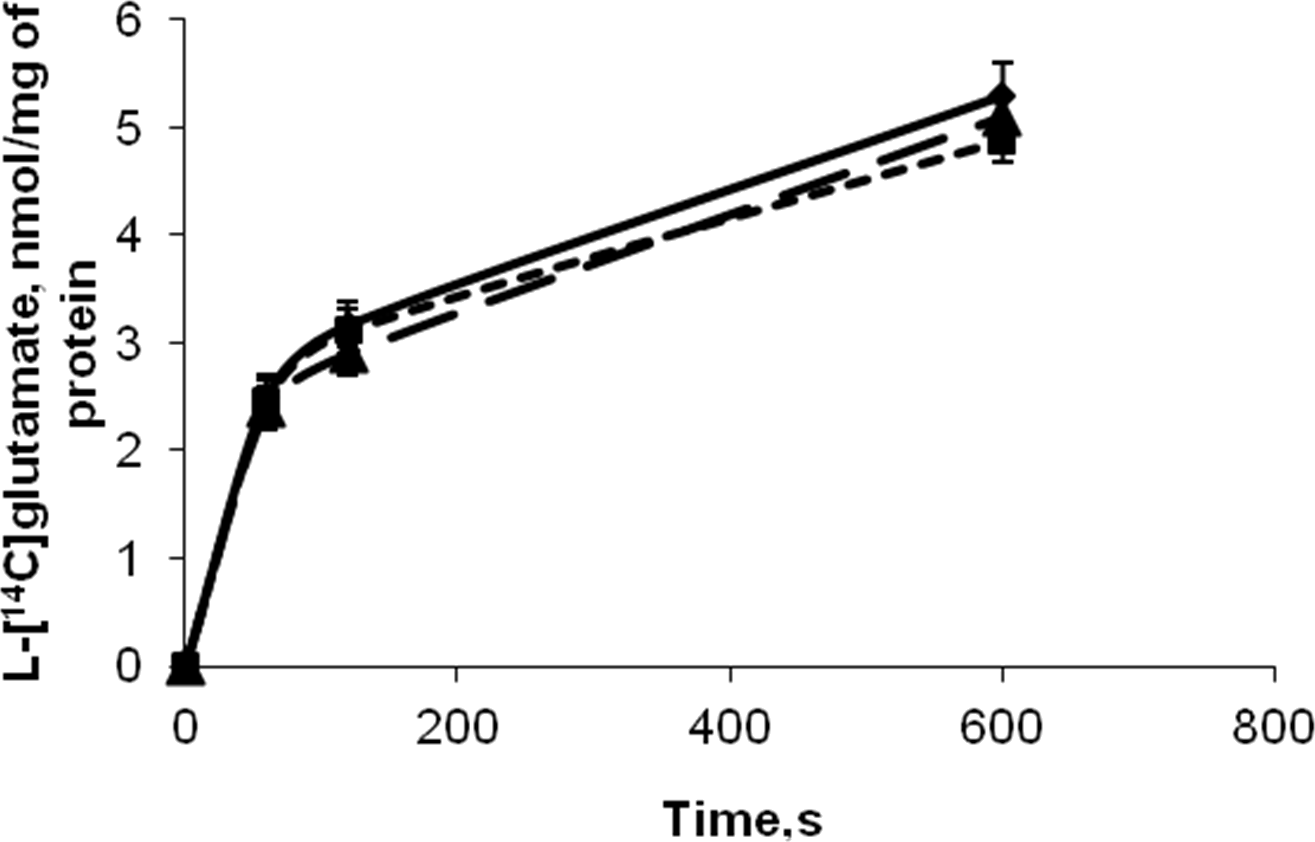
Time course of uptake of L-[^14^C]glutamate by control synaptosomes (solid line); synaptosomes in presence of uncoated γ-Fe_2_O_3_ nanoparticles (dashed line) and D-mannose-coated γ-Fe_2_O_3_ nanoparticles (dotted line). Uptake was initiated by the addition of L-[^14^C]glutamate to synaptosomes, after incubation the samples were rapidly sedimented and radioactivity was determined as described in section Experimental. Data represent mean ± SEM of three independent experiments.

Recently, it was shown that D-mannose-coated γ-Fe_2_O_3_ nanoparticles can penetrate cell membranes and can be internalized by the mesenchymal cells [12]. To assess the possibility of using these nanoparticles for manipulation of brain nerve terminals by an external magnetic field, their effect on the key characteristics of glutamatergic neurotransmission was analyzed. Isolated rat brain nerve terminals (synaptosomes) were used in the experiments. Synaptosomes are one of the best systems to explore the relationship between the structure of a protein, its biochemical properties, and physiological role [18]. Synaptosomes retain all characteristics of intact nerve terminals, that is, the ability to maintain the membrane potential, and to accomplish glutamate uptake, exocytosis, etc. As shown in Figure 3, incubation of D-mannose-coated and uncoated γ-Fe_2_O_3_ nanoparticles with synaptosomes for 10 min did not cause significant changes in the initial velocity of high affinity Na^+^-dependent L-[^14^C]glutamate uptake and the accumulation of L-[^14^C]glutamate by synaptosomes. The initial velocity of L-[^14^C]glutamate uptake by nerve terminals was equal to 2.5 ± 0.2 nmol·min^−1^·mg^−1^ protein in the control experiments, 2.45 ± 0.2 nmol·min^−1^·mg^−1^ protein in the presence of D-mannose-coated γ-Fe_2_O_3_ nanoparticles, and 2.4 ± 0.2 nmol·min^−1^·mg^−1^ protein in the presence of uncoated nanoparticles.

### Extracellular level and tonic release of glutamate from nerve terminals in presence of D-mannose-coated γ-Fe_2_O_3_ nanoparticles

**Figure 4:**
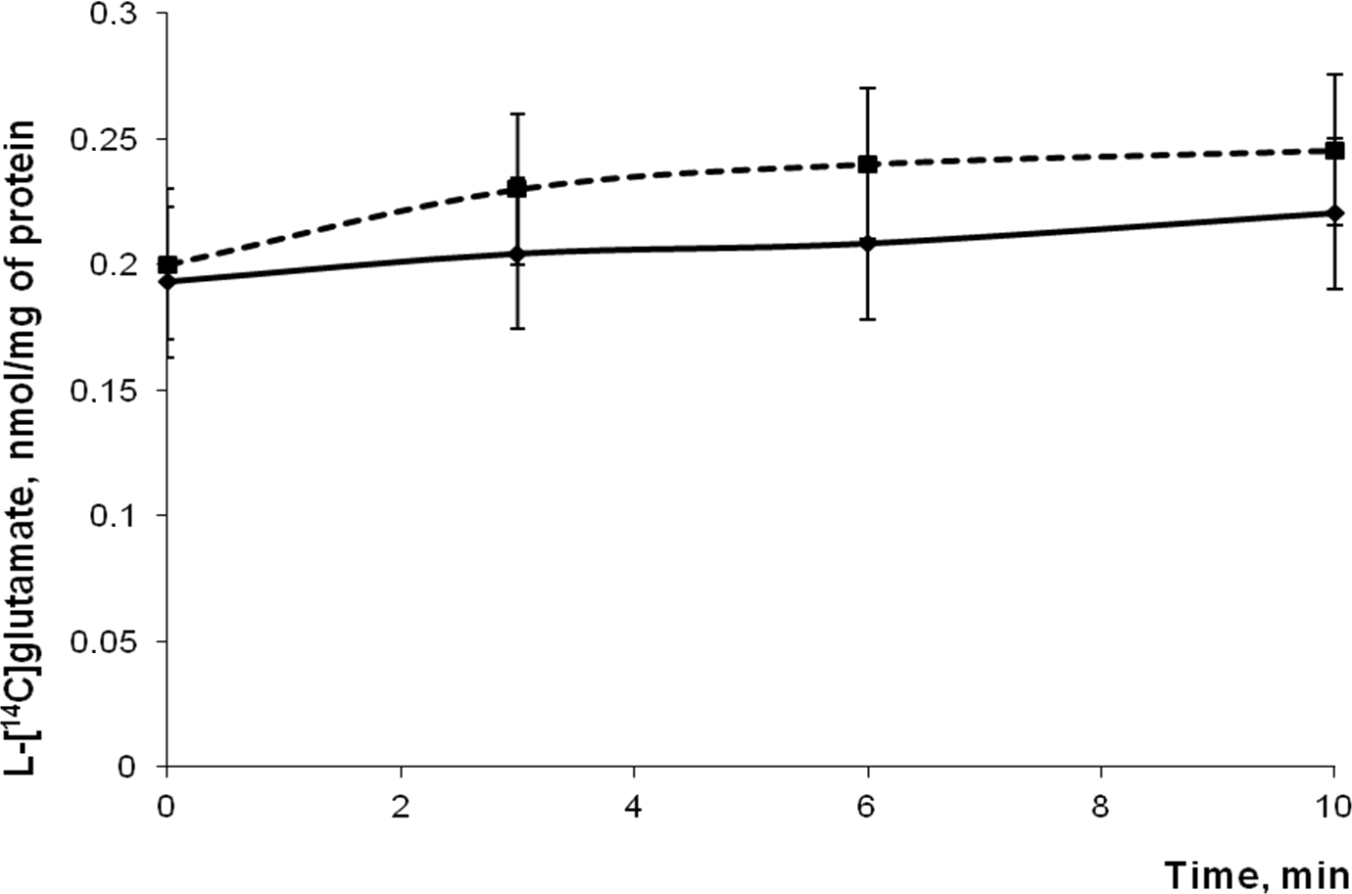
Ambient level of L-[^14^C]glutamate in the control nerve terminals (solid line), and in the presence of D-mannose-coated γ-Fe_2_O_3_ nanoparticles (250 µg/mL) (dotted line). After loading with L-[^14^C]glutamate (1 nmol/mg of protein, 238 µCi/mmol), aliquots of synaptosomes were collected at different time intervals, centrifuged, and the radioactivity was determined as described in section Experimental. Total synaptosomal L-[^14^C]glutamate content was equal to 200,000 ± 15,000 cpm/mg protein. Data are means ± SEM of five independent experiments, each performed in triplicate.

As it was stated in the introduction, a certain level of ambient glutamate is very important for normal synaptic transmission, whereas even a small increase in this level causes neurotoxicity. Nowadays, it is suggested that in unstimulated nerve terminals glutamate enriches the extracellular space by spontaneous exocytosis, and through swelling-activated anion channels, cysteine/glutamate exchange, trans-membrane diffusion and volume-sensitive Cl^−^ channels [19–21]. In this set of experiments, the extracellular level and unstimulated release of glutamate from nerve terminals in the presence of D-mannose-coated γ-Fe_2_O_3_ nanoparticles was analyzed. Ambient level of L-[^14^C]glutamate was measured after the addition of D-mannose-coated γ-Fe_2_O_3_ nanoparticles at concentration of 250 µg/mL to the incubation medium containing the synaptosomes. As shown in Figure 4, no significant changes in the extracellular level of L-[^14^C]glutamate in the synaptosomal suspension were found. After 6 min of incubation it was 0.204 ± 0.03 nmol of L-[^14^C]glutamate/mg of protein in the control and 0.230 ± 0.03 nmol of L-[^14^C]glutamate/mg of protein in the presence of nanoparticles. Tonic release of L-[^14^C]glutamate from nerve terminals in the presence of D-mannose-coated γ-Fe_2_O_3_ nanoparticles was analyzed. It was not significantly changed in the presence of nanoparticles as compared to the control synaptosomes and amounted to 0.027 ± 0.005 nmol/mg of protein in the control and 0.045 ± 0.005 nmol/mg of protein after incubation of synaptosomes with nanoparticles for 10 min. Uncoated γ-Fe_2_O_3_ nanoparticles also did not influence the extracellular level and tonic release of L-[^14^C]glutamate from synaptosomes (this data practically does not differ from those presented in Figure 4, and thus, is not shown).

### Membrane potential of nerve terminals in the presence of D-mannose-coated γ-Fe_2_O_3_ nanoparticles

**Figure 5:**
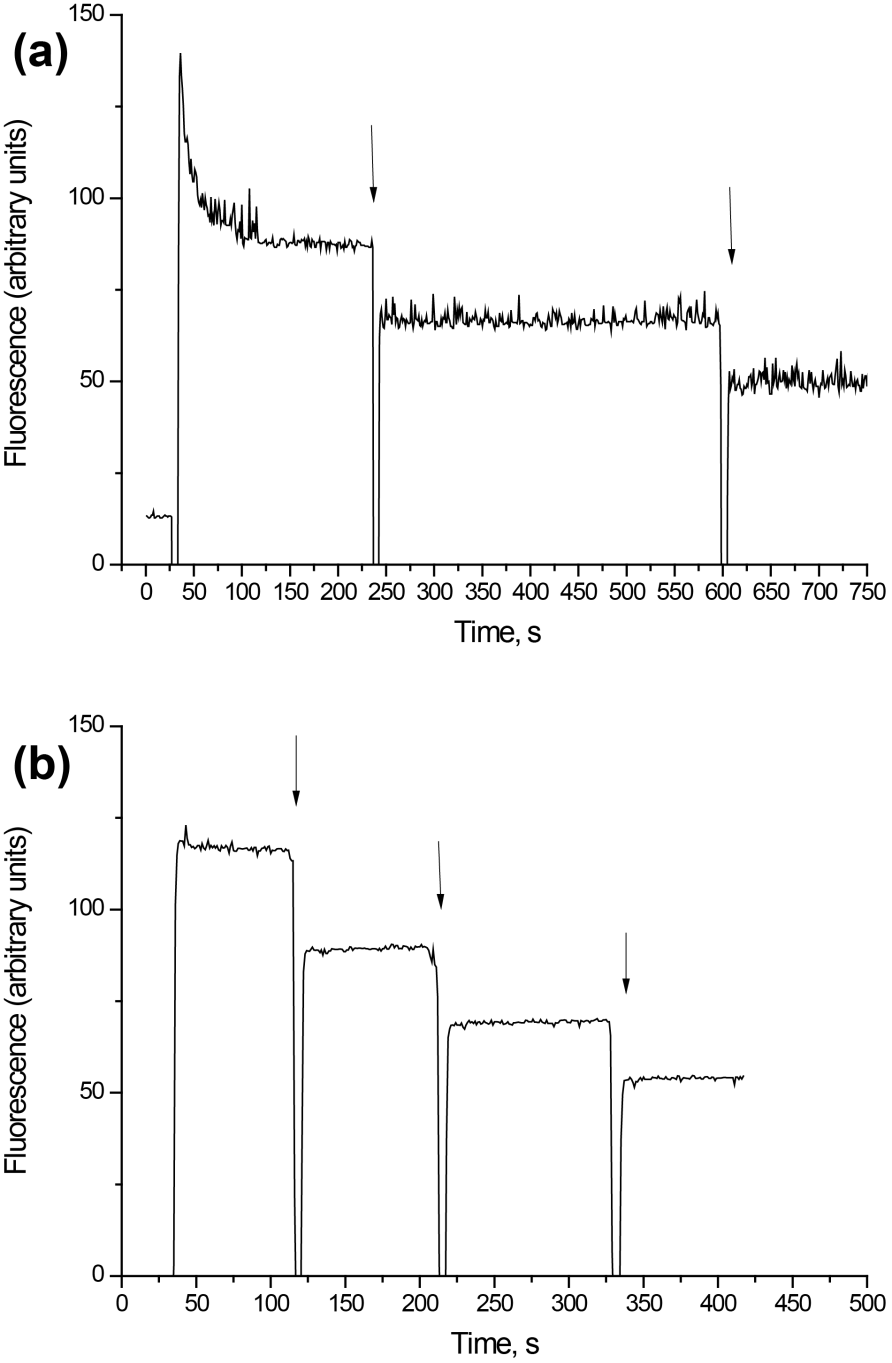
(a) Membrane potential of the synaptosomes after the addition of D-mannose-coated γ-Fe_2_O_3_ nanoparticles. The suspension of the synaptosomes was equilibrated with rhodamine 6G (0.5 µM); when the steady level of the dye fluorescence had been reached, nanoparticles (250 µg/mL) were added (arrows). (b) Rhodamine 6G fluorescence after the addition of D-mannose-coated γ-Fe_2_O_3_ nanoparticles (without synaptosomes). Each trace represents four experiments performed with different preparations.

Measurement of the plasma membrane potential *E*_m_ was performed by using rhodamine 6G, which binds to negative charges of the membranes. As shown in Figure 5a, addition of synaptosomal suspension to the medium containing rhodamine 6G was accompanied by a partial decrease in fluorescence owing to the binding of the dye to the plasma membrane. The membrane potential index at the steady state level, *F*_st_, was achieved after 3 min. Application of D-mannose-coated γ-Fe_2_O_3_ nanoparticles at a concentration of 250 µg/mL caused an acute decrease in the fluorescence signal (Figure 5a). This decrease was also registered in the absence of synaptosomes (Figure 5b), and could hence be subtracted from the experimental data. The resulting curve demonstrates that D-mannose-coated γ-Fe_2_O_3_ nanoparticles did not influence the fluorescence of rhodamine 6G, which reflects the absence of a depolarization of the plasma membrane of the nerve terminals. Uncoated γ-Fe_2_O_3_ nanoparticles also did not alter the membrane potential of synaptosomes (data not shown).

### Proton gradient of synaptic vesicles in the presence of D-mannose-coated γ-Fe_2_O_3_ nanoparticles

**Figure 6:**
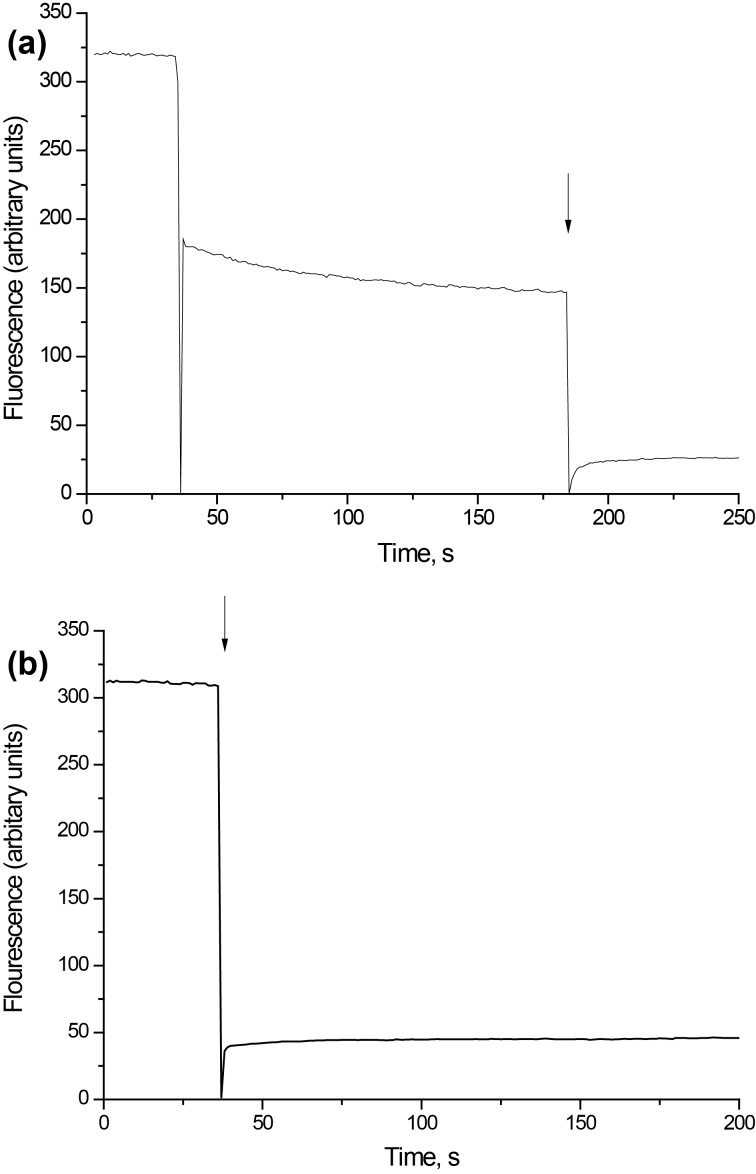
(a) Acidification of the synaptosomes in the presence of D-mannose-coated γ-Fe_2_O_3_ nanoparticles. The synaptosomes were equilibrated with acridine orange (5 µM); when the steady level of the dye fluorescence had been reached, nanoparticles (250 µg/mL) were added (arrow). (b) Acridine orange fluorescence after the addition of D-mannose-coated γ-Fe_2_O_3_ nanoparticles (without synaptosomes). Each trace represents four experiments performed with different preparations.

After reaching the cytosol, glutamate is accumulated in synaptic vesicles by vesicular glutamate transporters, which use the proton electrochemical gradient as a driving force. Uptake of glutamate by plasma membrane transporters depends on the proton gradient of synaptic vesicles. In these experiments, synaptic vesicle acidification as a component of electrochemical proton gradient (∆μ_H+_) was measured by using acridine orange, which is selectively accumulated by the acidic compartments of synaptosomes, that is synaptic vesicles. As shown in Figure 6, the addition of acridine orange to the synaptosomes was accompanied by a partial quenching of the fluorescence signal because of the dye accumulation in synaptic vesicles. After loading with acridine orange, D-mannose-coated γ-Fe_2_O_3_ nanoparticles were added to the synaptosomes. The addition of D-mannose-coated γ-Fe_2_O_3_ nanoparticles at a concentration of 250 µg/mL caused an acute decrease of the fluorescence (Figure 6a). This decrease was also registered in the absence of synaptosomes, and consequently the subtraction of fluorescence baseline was done similarly to that described in the previous subchapter (Figure 6b). The resulting curve demonstrated that nanoparticles did not influence the fluorescence signal of acridine orange. This data indicated that synaptic vesicles retained their proton gradient in the presence of D-mannose-coated γ-Fe_2_O_3_ nanoparticles. Uncoated γ-Fe_2_O_3_ nanoparticles also did not change synaptic vesicle acidification (data not shown). It can be thus concluded that both D-mannose-coated and uncoated γ-Fe_2_O_3_ nanoparticles did not significantly affect functions of nerve terminal and key characteristics of glutamatergic transmission.

### Manipulation of nerve terminals by an external magnetic field in the presence of D-mannose-coated γ-Fe_2_O_3_ nanoparticles

Manipulation of living cells by application of an external magnetic field, which penetrates through biological matter, receives great attention and can open new possibilities in biotechnology and medicine. In the next sets of the experiments, an external magnetic field (250 mT, gradient 5.5 Т/m) was applied to the bottom or side of tubes containing synaptosomal suspension (1.4 mL, 1 mg protein/mL). Distribution near the magnet area of control synaptosomes and synaptosomes incubated with nanoparticles at a concentration of 250 µg/mL for 5 min was assessed. Visually, synaptosomes preliminary incubated with D-mannose-coated γ-Fe_2_O_3_ nanoparticles moved immediately to the external magnet. Simultaneously, the opaque synaptosomal suspension became transparent in the part of the tube that was opposite to the magnet.

As it was stated above, the uptake, the extracellular level and the tonic release of glutamate in nerve terminals was not altered in the presence of D-mannose-coated γ-Fe_2_O_3_ nanoparticles, therefore L-[^14^C]glutamate-loaded nerve terminals can be used for quantitative assessment of their movement in response to the application of an external magnetic field. The amount of the radioactive label incorporated in the synaptosomes was calculated as percentage of the total amount of the label in the suspensions before and after the application of an external magnetic field. The synaptosomes were taken both from distant and proximal zone to the magnet, and then rapidly sedimented using a microcentrifuge and radioactivity in pellets was measured by liquid scintillation counting. The extracellular level of L-[^14^C]glutamate, which was similar for all probes, was subtracted.

**Figure 7:**
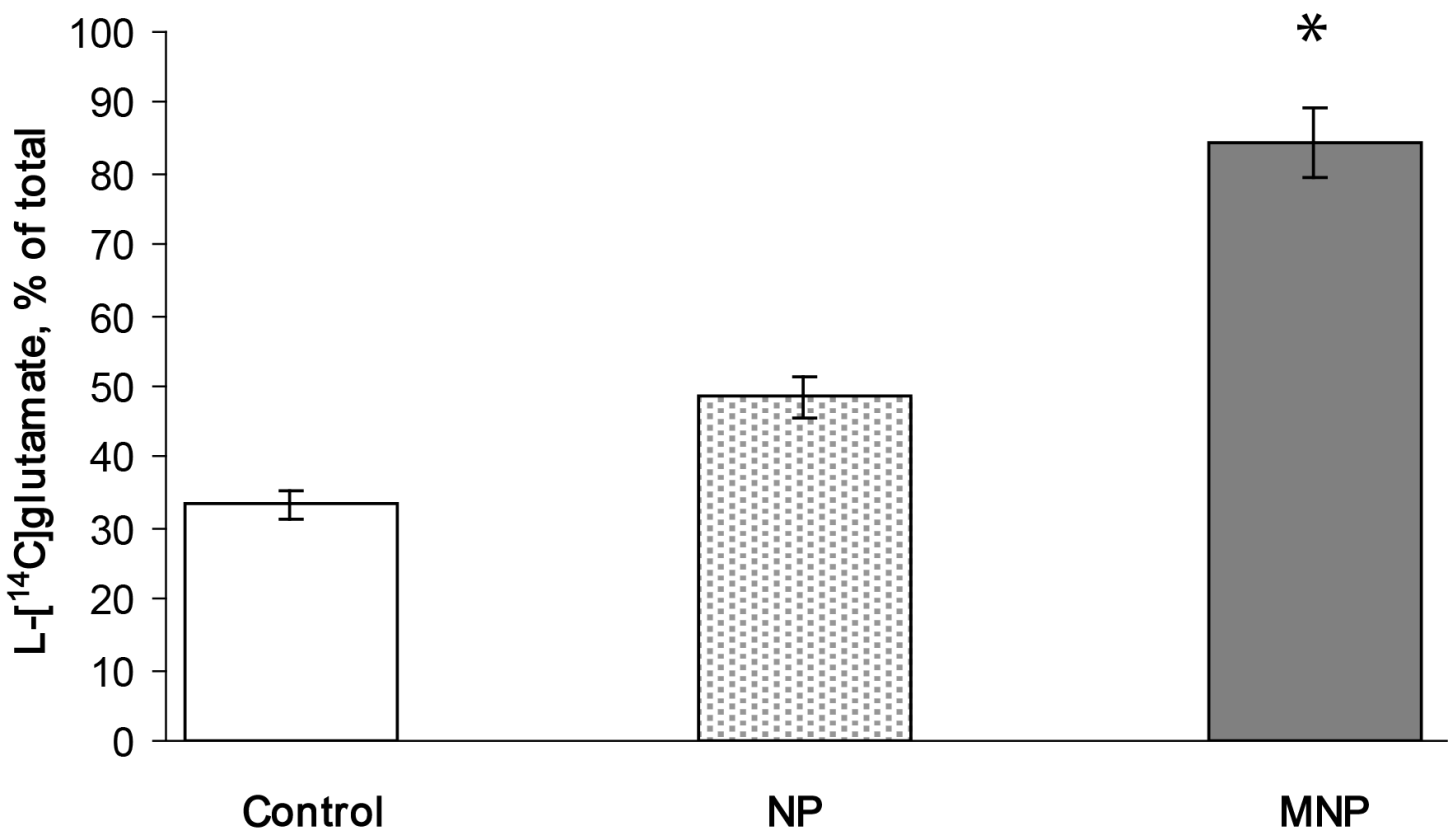
Movement of L-[^14^C]glutamate-containing synaptosomes to the magnet. Control (transparent column): synaptosomes in the absence of nanoparticles, NP (dotted column): synaptosomes in the presence of uncoated γ-Fe_2_O_3_ nanoparticles, MNP (grey column): synaptosomes in the presence of D-mannose-coated γ-Fe_2_O_3_ nanoparticles. The synaptosomes were pre-loaded with L-[^14^C]glutamate (1 nmol/mg of protein, 238 mCi/mmol), then uncoated or D-mannose-coated nanoparticles were added to synaptosomes and the amount of L-[^14^C]glutamate the aliquots of synaptosomal suspension in the vicinity of application of magnetic field was determined after rapid sedimentation by using a microcentrifuge (20 s at 10,000*g*). Total synaptosomal L-[^14^C]glutamate content was equal to 200,000 ± 15,000 cpm/mg protein. Data are means ± SEM of five independent experiments, each performed in triplicate. *Р ≤ 0.05, Student's *t*-test.

Results demonstrated that L-[^14^C]glutamate-loaded synaptosomes moved to the magnet and were concentrated in the magnet area. The amount of L-[^14^C]glutamate-loaded synaptosomes in the magnet area was 33.5 ± 3.0% of total label in control, 48.6 ± 3.0% of total label after the application of uncoated γ-Fe_2_O_3_ nanoparticles and 84.3 ± 5.0% of total label after the application of D-mannose-coated γ-Fe_2_O_3_ nanoparticles (*Р* ≤ 0.05, Student's *t*-test, *n* = 5) (Figure 7). Therefore, almost all D-mannose-coated γ-Fe_2_O_3_-labeled synaptosomes moved to the magnet.

These results were confirmed by the analysis of the protein concentration of synaptosomal suspension in the magnet area. It should be noted that synaptosomal preparations contained different types of nerve terminals, e.g., glutamatergic and GABA-ergic terminals, and they could be also contaminated by fragments of glial cells and inactive synaptosomes. Therefore, measurements based on the assessment of the synaptosomal protein concentration only, can bring some inaccuracy in the experimental data. Using L-[^14^C]glutamate in the experiments allowed us to analyze the movement of active glutamatergic nerve terminals exclusively. Clarifying involvement of the ionic strength in nanoparticle-synaptosome interaction, the effect of elevated concentrations of K^+^ on the movement of synaptosomes labeled with D-mannose coated γ-Fe_2_O_3_ nanoparticles to the magnet was investigated. KCl at a concentration of 35 mM was typically used for the depolarization of the plasma membrane of synaptosomes [13]. In this context, an increased extracellular level of L-[^14^C]glutamate in the presence of 35 mM KCl was taken into consideration. As a result, a decrease in the label by approximately 8% in vicinity of the magnet was revealed after 5 min incubation of synaptosomes with D-mannose-coated γ-Fe_2_O_3_ nanoparticles in the presence of 35 mM KCl. Therefore, it can be concluded that we obtained normally functioning synaptosomes labeled with D-mannose-coated γ-Fe_2_O_3_ nanoparticles, which can be easily manipulated by an external magnetic field.

## Discussion

A growing number of patients suffering from neurological and neurodegenerative disorders represent a heavy burden to the society around the world. Development of new methodological approaches for the manipulation of nerve cells by an external magnetic field can open new possibilities in disorder treatment. Methods based on the remote manipulation of magnetic nanoparticle-labeled cells by magnetic fields are receiving a great attention essentially because magnetic fields are not screened by biological matter and culture media.

Human nerve cells responsible for brain function are delicate biological objects vulnerable to ischemia, and other external factors. Due to ability to pass through biological membranes nanoparticles can produce toxic effects on nerve cells [2] but have a potential to cross the blood brain barrier that may open new ways for drug delivery into the brain [22]. Cobalt ferrite nanoparticles coated by silica, with a size of 50 nm, were found in the brain after being administered via an intravenous injection in mice [23]. After exposure of mice to TiO_2_ nanoparticles, they were found in the brain [24]. Nanoparticles instilled intranasally may target the central nervous system by formation of deposits of the particles on the olfactory mucosa of the nasopharyngeal region of the respiratory tract and their subsequent translocation via the olfactory nerve. One-fifth of the nanoparticles deposited on the olfactory mucosa can move to the olfactory bulb of rat brain providing a portal for entry into the central nervous system circumventing the blood–brain barrier [25]. In an in vitro model, it was shown that the ability of superparamagnetic iron oxide nanoparticles to penetrate the blood–brain barrier increased significantly in the presence of an external magnetic force. Therefore, particles can be transported through the blood–brain barrier and taken up by astrocytes, while they do not affect the viability of the endothelial cells [26]. On the cellular level, nanoparticles can pass through the plasma membrane of the cells by means of endocytosis [27]. Opposite point of view argues that uptake of nanoparticles into the cells does not occur by endocytic processes, but rather by diffusion or adhesive interactions [28].

In this study, we have presented a new approach of manipulation of isolated brain nerve terminals by an external magnetic field using D-mannose-coated γ-Fe_2_O_3_ nanoparticles. It was shown that nerve terminals after preliminary incubation with D-mannose-coated γ-Fe_2_O_3_ particles can be moved to the magnet more effectively in comparison with those labeled with uncoated ones (Figure 7). This fact has a great potential for cell investigation, cell separation and controlled movement, as well as for diagnostic, drug applications and regeneration of injured neurons. Integration of this methodological approach with heating by alternating magnetic fields to destroy cells could provide an additional tool for medical treatment. The mechanism by which D-mannose-coated γ-Fe_2_O_3_ nanoparticles interacted with nerve terminals should be further elucidated. However, it was demonstrated that elevated ionic strength (35 mM KCl) attenuated the effectiveness of manipulation of nerve terminals with D-mannose-coated γ-Fe_2_O_3_ nanoparticles by 8%.

The question rose whether the application of D-mannose-coated γ-Fe_2_O_3_ nanoparticles was accompanied with functional alterations in nerve terminals. Nanoparticles are able to penetrate the plasma membrane, and directly influence intracellular proteins, organelles, and DNA within the cells that may cause the development of neurotoxic effects [2,28]. It was shown that TiO_2_ nanoparticles induced an increase in glial fibrillary acidic protein, producing positive astrocytes in the CA4 region, which correlated with higher Ti contents in the hippocampus region. This lead to oxidative stress in the brain of exposed mice, an increase in the activity of catalase, and the excessive release of glutamic acid and nitric oxide [29].

In this study, neurotoxic effects of D-mannose-coated γ-Fe_2_O_3_ nanoparticles have been assessed. We clearly demonstrated that application of D-mannose-coated γ-Fe_2_O_3_ nanoparticles did not affect high-affinity Na^+^-dependent uptake, tonic release and the extracellular level of L-[^14^C]glutamate in rat brain nerve terminals (Figure 3 and Figure 4). Also, the potential of the plasma membrane of the nerve terminals and acidification of synaptic vesicles were not altered by D-mannose-coated γ-Fe_2_O_3_ nanoparticles, which was shown by fluorimetric measurements with potential-sensitive dye rhodamine 6G and pH-sensitive dye acridine orange (Figure 5 and Figure 6). Therefore, we demonstrated that D-mannose-coated γ-Fe_2_O_3_ nanoparticles added to nerve terminals did not affect the key characteristics of glutamatergic neurotransmission and retained unchanged functionality of nerve terminals, so they are non-neurotoxic when applied acutely. Using these nanoparticles, the possibility to manipulate nerve terminals by an external magnetic field was shown. In other words, functionally active nerve terminals labeled with γ-Fe_2_O_3_nanoparticles coated by D-mannose were obtained.

## Experimental

### Materials

FeCl_2_·4H_2_O and FeCl_3_·6H_2_O were purchased from Sigma-Aldrich (Steinheim, Germany), sodium hypochlorite solution (NaOCl) from Bochemie (Bohumín, Czech Republic), and sodium citrate dihydrate from Lachema (Brno, Czech Republic). D-mannose was from Acros (Geel, Belgium). Ethylene glycol tetraacetic acid (EGTA) and HEPES were purchased from Sigma-Aldrich (USA). Acridine orange and rhodamine 6G were obtained from Molecular Probes (USA); Ficoll 400, L-[^14^C]glutamate, aqueous counting scintillant (ACS) were from Amersham (UK). Analytical grade salts were from Sigma-Aldrich (USA).

### Synthesis of D-mannose-coated superparamagnetic maghemite nanoparticles

An aqueous solution of 0.2 M FeCl_3_ (100 mL) was mixed with of 0.5 M NH_4_OH solution (94 mL; less than an equimolar amount) under sonication (450 Digital Sonifier; Branson Ultrasonics, Danbury, CT, USA) at 23 °C for 5 min to form colloidal Fe(OH)_3_. Aqueous 0.2 M FeCl_2_ (50 mL) was then added under sonication and the mixture was poured in an excess of 0.5 M NH_4_OH aqueous solution (350 mL) under a nitrogen atmosphere. The resulting coagualate of magnetite was left to grow for 45 min under nitrogen atmosphere, then magnetically separated and repeatedly (7–10×) washed (peptized) with Q-water to remove all impurities (including NH_4_Cl) remaining after the synthesis. Finally, 0.1 M sodium citrate (13 mL) was added under sonication, and the magnetite was oxidized by slow addition of 5% sodium hypochlorite solution (10 mL) to yield maghemite (γ-Fe_2_O_3_). The above described washing procedure was repeated with the resulting primary colloid, which was finally passed through a Millex HV syringe filter (0.45 µm membrane, 33 mm in diameter). Coating of colloidal iron oxide nanoparticles was achieved by the post-synthesis method [12]. Aqueous D-mannose (2 mL; concentration 128 mg/mL) was added dropwise under sonication to a portion of primary colloid containing 44 mg of iron oxide and finally diluted to a volume of 10 mL with ultrapure water. The obtained mixture was sonicated for 5 min and resulting D-mannose coated γ-Fe_2_O_3_ particles were used in biological experiments.

### Properties of particles

The particle shape, diameter and size distribution were recorded by a JEOL JEM 200 CX transmission electron microscope (TEM). The size was calculated by using the Atlas program (Tescan, Digital Microscopy Imaging, Brno, Czech Republic). The hydrodynamic diameter *D*_h_ (z-average) and polydispersity as a measure of the particle size distribution were determined by dynamic light scattering (DLS) with an Autosizer Lo-C (Malvern Instruments, UK). The modification of the nanoparticle surface with D-mannose was analyzed using a Nicolet Impact 400 Fourier transformation infrared (FTIR) spectrometer in water-purged surrounding with a DTGS detector. The spectra were measured by ATR spectroscopy with the Golden Gate^TM^ single reflection system (Specac Ltd., Orpington, UK) at 400–4000 cm^−1^.

### Ethics statement

Wistar male rats, 100–120 g body weight, were obtained from the vivarium of M. D. Strazhesko Institute of Cardiology, Medical Academy of Sciences of Ukraine. Animals were kept in animal facilities of the Palladin Institute of Biochemistry in accordance with the European guidelines and international laws and policies. They were housed in a quiet, temperature-controlled room (22–23 °C) and were provided with water and dry food pellets ad libitum. Before removing the brain, rats were decapitated. Experimental protocols were approved by the Animal Care and Use Committee of the Palladin Institute of Biochemistry (Protocol from 19/09-2011).

### Isolation of rat brain nerve terminals (synaptosomes)

Synaptosomes were prepared from rat brain as described in [30]. Cerebral hemispheres of decapitated animals were removed and homogenized rapidly in ice-cold 0.32 M sucrose, 5 mM HEPES–NaOH, pH 7.4, and 0.2 mM EDTA. The synaptosomes were prepared by differential and Ficoll-400 density gradient centrifugation of rat brain homogenate [31] with slight modifications [30]. All manipulations were performed at 4 °C. The synaptosomal suspensions were used in experiments during 2–4 h after isolation. The standard salt solution was oxygenated and contained (in mM): NaCl 126; KCl 5; MgCl_2_ 2.0; NaH_2_PO_4_ 1.0; HEPES 20, pH 7.4; and D-glucose 10. Ca^2+^-supplemented medium contained 2 mM CaCl_2_. The Ca^2+^-free medium contained 1 mM EGTA and no added Ca^2+^. Protein concentration was measured as described by Larson et al. [32].

### L-[^14^C]glutamate uptake by nerve terminals

The uptake of L-[^14^C]glutamate by synaptosomes was measured as described in [33]. The synaptosomal suspension (125 μL of the suspension, 0.2 mg of protein/mL) was pre-incubated in standard salt solution at 37 °C for 8 min, then magnetic nanoparticles (250 µg/mL; stock solution 4.4 mg/mL was diluted 18 times) were added to the synaptosomal suspension and incubated for 10 min. Uptake was initiated by the addition of 10 µM L-glutamate supplemented with 420 nM L-[^14^C]glutamate (0.1 µCi/mL), incubated at 37 °C during different time intervals (1, 2, 10 min) and then rapidly sedimented by using a microcentrifuge (20 s at 10,000*g*). L-[^14^C]glutamate uptake was determined as a decrease in radioactivity in aliquots of the supernatant (100 μL) and an increase in radioactivity of the pellet (SDS-treated) measured by liquid scintillation counting with ACS scintillation cocktail (1.5 mL).

### L-[^14^C]glutamate release from nerve terminals

The release of L-[^14^C]glutamate from the synaptosomes was measured as described in [34–35]. The synaptosomes were diluted in standard salt solution to reach concentration of 2 mg of protein/mL and after pre-incubation at 37 °C for 10 min they were loaded with L-[^14^C]glutamate (1 nmol/mg of protein, 238 mCi/mmol) in Ca^2+^-supplemented oxygenated standard salt solution at 37 °C for 10 min. After loading**,** suspension was washed with 10 volumes of ice-cold oxygenated standard salt solution; the pellet was resuspended in a solution to a final concentration of 1 mg protein/mL and immediately used for release experiments. Release of L-[^14^C]glutamate from the synaptosomes was performed in Ca^2+^-free incubation medium according to following method. Synaptosomal suspension (125 μL; 0.5 mg of protein/mL) was pre-incubated for 10 min, then the nanoparticles (250 µg/mL; stock solution 4.4 mg/mL was diluted 18 times) were added at 37 °C and incubated for different time intervals (0, 3, 6, 10 min) and rapidly sedimented by using a microcentrifuge (20 s at 10,000*g*). Release was measured in the aliquots of the supernatants (100 μL) by liquid scintillation counting with scintillation cocktail ACS (1.5 mL) and was expressed a percentage of total amount of radiolabeled neurotransmitter incorporated. Release of the neurotransmitter from the synaptosomes incubated in Ca^2+^-free media without stimulating agents was used for assay of unstimulated (tonic) release.

### Measurement of synaptosomal plasma membrane potential (*E*_m_)

The membrane potential was measured as described in [36–37] by using rhodamine 6G (0.5 µM) which binds to the plasma membrane. The suspension of synaptosomes (0.2 mg/mL of final protein concentration) preincubated at 37 °C for 10 min was added to a stirred thermostatted cuvette. To estimate changes in the plasma membrane potential the ratio (*F*) as index of membrane potential was calculated according to Equation 1:

[1]
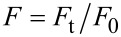


where *F*_0_ and *F*_t_ are fluorescence intensities of a fluorescent dye in the absence and presence of the synaptosomes, respectively. *F*_0_ was calculated by extrapolation of the exponential decay function to *t* = 0.

Rhodamine 6G fluorescence measurements were carried by using a Hitachi MPF-4 spectrofluorimeter at 528 nm (excitation) and 551 nm (emission) wavelengths (slit bands 5 nm each).

### Measurements of synaptic vesicle acidification in synaptosomes

The acidification of synaptic vesicles in synaptosomes was monitored by using acridine orange as described in [38–39]. Fluorescence changes were measured by using a Hitachi MPF-4 spectrofluorimeter at excitation and emission wavelengths of 490 and 530 nm, respectively (slit bands 5 nm each). Reaction was started by the addition of acridine orange (final concentration 5 μM) to synaptosomal suspension (0.2 mg/mL of final protein concentration) preincubated in a stirred thermostatted cuvette at 30 °C for 10 min. The equilibrium level of dye fluorescence was achieved after 3 min. Fluorescence (*F*) was determined according to Equation 1.

### Manipulation of D-mannose-coated γ-Fe_2_O_3_-labeled synaptosomes by an external magnetic field

Synaptosomes were diluted in standard salt solution to concentration of 2 mg of protein/mL and after pre-incubation at 37 °C for 10 min they were loaded with L-[^14^C]glutamate (1 nmol/mg of protein, 238 mCi/mmol) in Ca^2+^-supplemented oxygenated standard salt solution at 37 °C for 10 min. After loading, the suspension was washed with 10 volumes of ice-cold oxygenated standard salt solution; the pellet was resuspended in the solution to a final concentration of 1 mg protein/mL and immediately used for release experiments.

The total amount of label incorporated in synaptosomes was calculated before application of an external magnetic field as follows. Suspension of labelled synaptosomes (125 μL) was rapidly sedimented by using a microcentrifuge (20 s at 10,000*g*). The total amount of label incorporated in synaptosomes was measured in the aliquots of the supernatants (100 μL) by liquid scintillation counting with ACS scintillation cocktail (1.5 mL) and was expressed as a percentage of total amount of radiolabeled neurotransmitter incorporated. An external magnetic field (250 mT, gradient 5.5 Т/m) was applied at the bottom or the side of tubes with synaptosomal suspension (1.4 mL) in the absence of nanoparticles (control) and to synaptosomes incubated with the nanoparticles (250 µg/mL; stock solution 4.4 mg/mL was diluted 18 times) for 5 min. The suspension (125 μL) was taken from both the distant and the proximal zone of the magnetic field, then rapidly sedimented by using a microcentrifuge (20 s at 10,000*g*), and the radioactivity in pellets was measured by liquid scintillation counting.

### Statistical analysis

The results were expressed as means ± S.E.M. of *n* independent experiments. The difference between two groups was compared by two-tailed Student's *t*-test. The differences were considered significant, when *Р* ≤ 0.05.

## References

[1] Yang Z, Liu Z W, Allaker R P, Reip P, Oxford J, Ahmad Z, Ren G (2010). J R Soc Interface.

[2] Brooking J, Davis S S, Illum L (2001). J Drug Targeting.

[3] Laurent S, Mahmoudi M (2011). Int J Mol Epidemiol Genet.

[4] Zhang Y, Zhang J (2005). J Colloid Interface Sci.

[5] Yeh T-C, Zhang W, Ildstad S T, Ho C (1993). Magn Reson Med.

[6] Yeh T-C, Zhang W, Ildstad S T, Ho C (1995). Magn Reson Med.

[7] Modo M, Cash D, Mellodew K, Williams S C R, Fraser S E, Meade T J, Price J, Hodges H (2002). NeuroImage.

[8] Jendelová P, Herynek V, De Croos J, Glogarova K, Andersson B, Hajek M, Sykova E (2003). Magn Reson Med.

[9] Jendelová P, Herynek V, Urdzíková L, Glogarová K, Kroupová J, Andersson B, Bryja V, Burian M, Hájek M, Syková E (2004). J Neurosci Res.

[10] Irache J M, Salman H H, Gamazo C, Espuelas S (2008). Expert Opin Drug Delivery.

[11] Labský J (2003). Biomaterials.

[12] Horák D, Babič M, Jendelová P, Herynek V, Trchová M, Pientka Z, Pollert E, Hájek M, Syková E (2007). Bioconjugate Chem.

[13] Borisova T, Sivko R, Borysov A, Krisanova N (2010). Cell Mol Neurobiol.

[14] Zoccarato F, Cavallini L, Alexandre A (1999). J Neurochem.

[15] Pollert E, Knížek K, Maryško M, Závěta K, Lančok A, Boháček J, Horák D, Babič M (2006). J Magn Magn Mater.

[16] Tocchio A, Horák D, Babic M, Trchová M, Veverka M, Beneš M J, Šlouf M, Fojtík A (2009). J Polym Sci, Part A: Polym Chem.

[17] Koppel D E (1972). J Chem Phys.

[18] Südhof T C (2004). Annu Rev Neurosci.

[19] Cavelier P, Attwell D (2005). J Physiol.

[20] Jabaudon D, Shimamoto K, Yasuda-Kamatani Y, Gähwiler B H, Gerber U (1999). Proc Natl Acad Sci U S A.

[21] Rutledge E M, Aschner M, Kimelberg H K (1998). Am J Physiol.

[22] De Jong W H, Borm P J A (2008). Int J Nanomed.

[23] Kim J S, Yoon T J, Yu K N, Kim B G, Park S J, Kim H W, Lee K H, Park S B, Lee J K, Cho M H (2006). Toxicol Sci.

[24] Takeda K, Suzuki K, Ishihara A, Kubo-Irie M, Fujimoto R, Tabata M, Oshio S, Nihei Y, Ihara T, Sugamata M (2009). J Health Sci.

[25] Oberdörster G, Sharp Z, Atudorei V, Elder A, Gelein R, Kreyling W, Cox C (2004). Inhalation Toxicol.

[26] Thomsen L B, Linemann T, Pondman K M, Lichota J, Kim K S, Pieters R J, Visser G M, Moos T (2013). ACS Chem Neurosci.

[27] Xia T, Kovochich M, Liong M, Zink J I, Nel A E (2008). ACS Nano.

[28] Geiser M, Rothen-Rutishauser B, Kapp N, Schürch S, Kreyling W, Schulz H, Semmler M, Im Hof V, Heyder J, Gehr P (2005). Environ Health Perspect.

[29] Wang J, Chen C, Liu Y, Jiao F, Li W, Lao F, Li Y, Li B, Ge C, Zhou G (2008). Toxicol Lett.

[30] Borisova T A, Krisanova N V (2008). Adv Space Res.

[31] Cotman C W (1974). Methods Enzymol.

[32] Larson E, Howlett B, Jagendorf A (1986). Anal Biochem.

[33] Borisova T (2013). Cholesterol and presynaptic glutamate transport in the brain.

[34] Krisanova N V, Trikash I O, Borisova T A (2009). Neurochem Int.

[35] Krisanova N, Sivko R, Kasatkina L, Borisova T (2012). Biochim Biophys Acta, Mol Basis Dis.

[36] Kasatkina L, Borisova T (2010). Neurochem Int.

[37] Kasatkina L, Borisova T A (2013). Int J Biochem Cell Biol.

[38] Borisova T, Kasatkina L, Ostapchenko L (2011). Neurochem Int.

[39] Borisova T, Krisanova N, Sivko R, Kasatkina L, Borysov A, Griffin S, Wireman M (2011). Neurochem Int.

